# Prevalence and Associated Factors of Hypomineralised Second Primary Molars in 3–5-Year-Old Children in Aydın, Türkiye

**DOI:** 10.3290/j.ohpd.c_2773

**Published:** 2026-08-06

**Authors:** Melis Bahar Akyıldız Çelik, Yiğit Karaoğlu, Özgün Öztürk, Ezgi Taşpınar, Işıl Sönmez

**Affiliations:** a Melis Bahar Akyıldız Çelik Specialist in Paediatric Dentistry, Department of Paediatric Dentistry, Faculty of Dentistry, Aydın Adnan Menderes University, Aydın, Turkey. Contributed to the conception, design, and project management, calibration of the examiner, and drafted the manuscript.; b Yiğit Karaoğlu PhD student, Department of Paediatric Dentistry, Faculty of Dentistry, Aydın Adnan Menderes University, Aydın, Turkey. Contributed to the conception, design, recording of the patient examination data and interpretation of the study, and drafted the manuscript.; c Özgün Öztürk PhD student, Department of Paediatric Dentistry, Faculty of Dentistry, Aydın Adnan Menderes University, Aydın, Turkey. Contributed to the conception and design, was responsible for patient examination, contributed to manuscript writing and interpretation of the study, and drafted the manuscript.; d Ezgi Taşpınar PhD student, Department of Paediatric Dentistry, Faculty of Dentistry, Aydın Adnan Menderes University, Aydın, Turkey. Contributed to the conception, design, recording of the patient examination data and interpretation of the study and drafted the manuscript.; e Işıl Sönmez Professor and Paediatric Dentistry Specialist, Department of Paediatric Dentistry, Faculty of Dentistry, Aydın Adnan Menderes University, Aydın, Turkey. Contributed to the conception, design, and project management, and also drafted the manuscript. All authors critically revised the manuscript, approved the final version, and agreed to be accountable for all aspects of the work.

**Keywords:** associated factors, hypomineralised second primary molars, prevalence, preschool children

## Abstract

**Purpose:**

To determine the prevalence and associated factors of hypomineralisation of second primary molars (HSPM) in children aged between 3 and 5 years, in Aydın.

**Methods and Materials:**

A total of 1035 children, aged between 3 and 5 years old, participated in the study from 16 randomly selected kindergartens across 5 districts of Aydın. HSPM findings were recorded using the modified EAPD MIH Decision Criteria Index. To determine the associated factors, a questionnaire consisting of questions about prenatal, perinatal, and postnatal factors was filled out by parents. Descriptive statistics and Pearson chi-square tests were used.

**Results:**

The mean age was 4.66 ± 0.53 years; the prevalence of HSPM was 4.4% at the patient level and 2.31% at the tooth level. White, cream, yellow or brown demarcated opacities were the most common lesion type (69.79%); the most affected jaw was the mandible (71.87%), and the mean number of affected teeth among the children with HSPM was 2.17 ± 0.99. Considering the associated factors of HSPM, maternal febrile illnesses during pregnancy and children’s febrile illnesses during perinatal and postnatal periods, along with nutritional supplement use, preterm birth, or breastfeeding duration, did not show statistical significance in HSPM development (*p* > 0.05).

**Conclusion:**

Results of this study showed that the prevalence of HSPM (4.4%) in Aydın was slightly lower than the worldwide prevalence (6.8%). HSPM had many possible causes; to more clearly determine the aetiology of HSPM, more research is needed.

Hypomineralised second primary molars (HSPM) are a qualitative enamel defect that occurs due to disturbances in the mineralisation of second primary molars during tooth development. Clinically, it is characterised by well-demarcated white, yellow, or brown opacities often similar in appearance to molar-incisor hypomineralisation (MIH). The condition and the term HSPM were described in second primary molars in the late 2000s using diagnostic approaches adapted from the European Academy of Paediatric Dentistry (EAPD) MIH criteria.^6,7,17^ Hypomineralised enamel has a porous and rough structure due to reduced mineral content. This structural weakness may lead to post-eruptive breakdown, particularly under masticatory forces. Such defects facilitate plaque and biofilm accumulation, complicate oral hygiene maintenance due to dentine hypersensitivity, and consequently increase the risk of dental caries.^5,11^

The first studies investigating the prevalence of HSPM based on these diagnostic criteria were conducted in the Netherlands.^6,7^ Globally, the reported prevalence of HSPM varies widely between countries and populations. A meta-analysis by McCarra et al^18^ also confirmed a broad range (0–41%), with a pooled global prevalence of 6.8%. Differences in prevalence across studies are thought to be related to variations in diagnostic indices and calibration methods^3^.

In Turkey, research on HSPM is limited. In a study conducted in Uşak province,^3^ the prevalence was reported as 3.5%. A more recent study^26^ conducted in Trabzon province reported that the prevalence of HSPM was found to be 6.8%.

Although strong evidence regarding the exact aetiology of HSPM is lacking, environmental factors are considered to play a more significant role than genetic factors.^24^ Potential risk factors discussed in the literature include prenatal, perinatal, and postnatal conditions such as maternal hypertension, low birth weight, prematurity, birth complications, nutritional status, vitamin deficiencies, infections, and chronic diseases.^3,14^

To date, no study investigating the prevalence and potential aetiological factors associated with HSPM has been identified in the region of Aydın. Therefore, the present study aimed to determine the prevalence of HSPM in children aged 3–5 years living in Aydın through intraoral examinations, and to identify potential associated factors using parental questionnaires. In addition, it aimed to assess the relationship between HSPM and dental caries according to the World Health Organization (WHO) caries diagnostic criteria.^27^

## METHODS AND MATERIALS

### Study Design and Settings

This study was designed as a descriptive, analytical-cross-sectional study. It was conducted in the province of Aydın during the 2023–2024 academic year.

### Ethical Approval and Obtaining Consent from Parents

Ethics committee approval for this study was obtained from the Non-Interventional Clinical Research Ethics Committee of the Faculty of Dentistry, Adnan Menderes University, Aydın (AADÜDHF 2023/07). It was conducted in accordance with the World Medical Association’s Declaration of Helsinki. As the study was to be conducted in schools, official permission was obtained from the Aydın Provincial National Education Research Planning Board prior to the study. All participants and their parents/legal guardians were informed, and consent forms were signed.

### Determining Sample Size and Randomisation

According to data from the Aydın Provincial Directorate of National Education, there were 61 public schools and 42 private schools. The number of children aged 3–5 attending preschool education in the province of Aydın was 15,367 in public schools and 2,513 in private schools, for a total of 17,880 (n = 17,880) in the 2023–2024 academic year.

To calculate the HSPM prevalence, the required sample size was determined using the Epi Info programme (Centers for Disease Control and Prevention (CDC) in Atlanta, Georgia (US)). Since the prevalence was unknown, it was assumed to be 50%, the design effect was set at 1.5, and the minimum number of participants required for the study at a 95% confidence interval was found to be 565.

A multi-stage sampling method was used to determine the districts and schools to be sampled. At the first stage, Aydın was divided into two strata: the provincial centre and the districts. Then, to determine which districts would be included in the study, the provincial centre was divided into four regions geographically (north, south, east, and west), and one district was selected from each region using a simple random method. As a result, a total of five districts (the provincial centre (Efeler) and the districts of Didim, Nazilli, Bozdoğan, and İncirliova) were included in the study.

At the second stage, schools were selected using a simple randomisation method based on the list of schools available in the districts. While reaching the sample size determined for each district, class selection was made from schools while maintaining the ratio of private school and public-school student numbers.

Four public and three private schools from the provincial centre (District of Efeler), one public school and one private school from the District of İncirliova, two public schools and one private school from the District of Nazilli, one public school and one private school from the District of Didim, and two public schools from the District of Bozdoğan were included in the study.

### Participants and Inclusion Criteria

Children aged 3–5 years and children whose parents consented to the study and completed the questionnaire were included in this study. Children whose legal guardians did not give consent or did not complete the questionnaire, children who were unable to adapt to the study, and children with mental and developmental disabilities or syndromes were excluded from this study.

### Intraoral Examinations and Measurements

Intraoral examinations were conducted in classrooms at schools using sterile stainless-steel mirror and probe sets under pen light illumination. Teeth were not dried, but excess saliva and plaque were removed from the surface of the teeth with the help of a sterile gauze pad to prepare the tooth surfaces for examination in accordance with the recommendations of Ghanim et al.^13^ All deciduous teeth present in the oral cavity, as well as all fully erupted first permanent molars, were clinically examined. The tooth surfaces were examined, and enamel defects were recorded according to the EAPD MIH/HSPM criteria.^13^ In the teeth diagnosed with HSPM, scores were assigned between 2 and 6 based on their clinical appearance. Limited opacities were given a score of 2, post-eruptive enamel breakdown 3, atypical restoration 4, atypical caries 5 and missing teeth due to MIH/HSPM 6. Lesion extensions were recorded (only after diagnosing MIH/HSPM) as follows: I: less than one-third of the tooth surface affected; II: at least one-third but less than two-thirds of the surface affected; III: at least two-thirds of the tooth surface affected. Also, carious lesions were recorded according to the World Health Organization (WHO) dft index.^27^

HSPM was differentiated from other enamel defects and early carious lesions using predefined clinical criteria. Enamel hypoplasia was identified by reduced enamel thickness, pits, grooves, or missing enamel, whereas HSPM showed normal enamel thickness with altered translucency. Fluorosis was distinguished by its diffuse, symmetrical appearance and poorly defined margins. Early carious lesions typically occurred in plaque-retentive areas and appeared translucent on wet surfaces but opaque white after air-drying, whereas hypomineralised defects remained opaque white even on wet surfaces. In contrast, HSPM presented as well-demarcated opacities, with or without post-eruptive enamel breakdown.

The clinical examination was performed by a single experienced researcher (ÖÖ) who was calibrated for the use of the HSPM index. In order to distinguish HSPM lesions from other mineralisation disorders and caries lesions, a reference examiner (B.M.A.) calibrated the researcher for the diagnosis and classification of HSPM and dental caries lesions prior to the clinical examination. For this purpose, a set of 60 photographs containing HSPM lesions of varying severity and different dental caries lesions was used. The reviewer evaluated the photographs twice with an interval of one week between evaluations, and the results were compared with the reference reviewer by obtaining intra-rater and inter-rater reliability through the Kappa coefficient. The intra- and inter- examiner Kappa coefficients for HSPM were 0.91 and 0.95, respectively.

The questionnaire was prepared by researchers, based on the literature, to evaluate the associated factors with HSPM.^9,14,15^ The questionnaire covered a range of areas, including demographic information (such as the child’s gender, age, parents’ level of education, and the family’s socioeconomic status), details of the child’s systemic health, and questions about the child’s prenatal, perinatal, and postnatal history (including the mother’s illnesses, medications and supplements during the pregnancy, birth complications, timing of delivery, child’s birth weight, duration of breastfeeding and the child’s illnesses up to the age one).

The questionnaire and an informed consent form were sent to parents one week before the intraoral examination day, and they were asked to fill it out and return it to the school on the day of examination.

### Statistical Methods

Statistical analyses were performed using SPSS version 22.0 (Statistical Package for the Social Sciences, IBM, Armonk, NY, USA). Descriptive statistics were calculated for all variables. Categorical variables were presented as frequencies and percentages, whereas quantitative variables were summarised as mean ± standard deviation or median and interquartile range (25th–75th percentile), as appropriate. The normality of quantitative variables was assessed using the Kolmogorov–Smirnov test. Differences between independent groups were analysed using the Mann–Whitney U test. Associations between categorical variables were examined using the chi-square test. Multivariable logistic regression analysis using the Forward (Wald) method was performed to determine the independent effects of the variables. A *p* value of less than 0.05 was considered statistically significant.

## RESULTS

**Fig 1:**
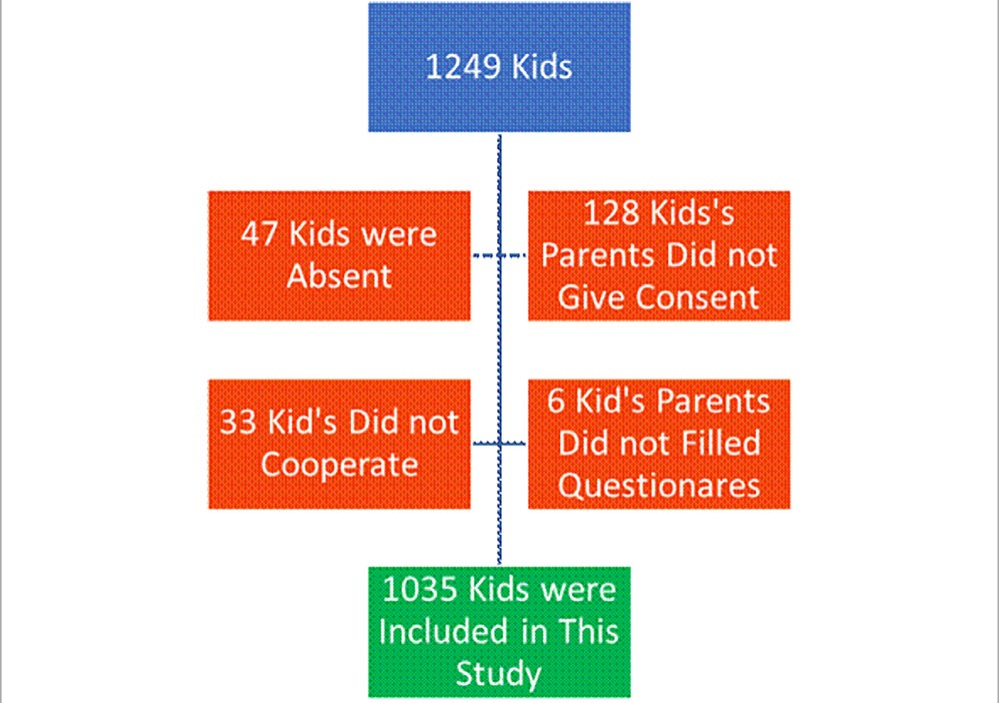
Participation flowchart.

To determine the prevalence of hypomineralised second primary molars (HSPM) in Aydın Province and to identify associated factors, 1,249 children were invited to participate from 16 schools across five districts. As shown in Figure 1, the participation rate was 82.9%, with 1,035 children included in the final analysis.

### Sociodemographic Characteristics

The mean age of the participating children was 4.66 ± 0.53 years. Regarding age distribution among affected children, 4.3% (n = 2) were 3 years old, 30.4% (n = 14) were 4 years old, and 65.2% (n = 30) were 5 years old. There seems to be no significant difference between ages for HSPM (*p* = 0.748). Of the total sample, 52.3% (n = 541) were female, and 47.7% (n = 494) were male. As shown in Table 1, there was no statistically significant difference in the prevalence of HSPM between females (43.5%, n = 20) and males (56.5%, n = 26) (*p* > 0.05). With respect to school type, 71.7% (n = 33) of affected children attended public schools, whereas 28.3% (n = 13) attended private schools. This difference was not statistically significant (*p* = 0.361). Similarly, no significant association was found between parents’ education level and the presence of HSPM (*p* = 0.749).

**Table 1:** Sociodemographic data

Variables	HSPM			
	Present N (%)	Absent N (%)	Total N (%)	*p* value
**Sex**				
Female	20 (3.7%)	521 (96.3%)	541 (52.3%)	0.231
Male	26 (5.3%)	468 (94.7%)	494 (47.7%)	
**Age**				
3	2 (6.7%)	28 (93.3%)	30 (2.9%)	0.748
4	14 (4.9%)	274 (95.1%)	288 (27.8%)	
5	30 (4.2%)	687 (95.8%)	717 (69.3%)	
**School type**				
Public	33 (4.1%)	777 (95.6%)	810 (78.3%)	0.275
Private	13 (5.8%)	212 (94.2%)	225 (21.7%)	
**Mother’s education level (n = 1030)**				
Less than high school	10 (5.8%)	163 (94.2%)	173 (16.8%)	0.546
High school	10 (3.6%)	269 (96.4%)	279 (27.1%)	
University	26 (4.5%)	552 (95.5%)	578 (56.1%)	
**Father’s education level (n = 1028)**				
Less than high school	11 (5.1%)	205 (94.9%)	215 (20.9%)	0.848
High school	14 (4.6%)	293 (95.4%)	306 (29.8%)	
University	21 (4.1%)	486 (95.9%)	507 (49.3%)	


### HSPM Prevalence and Distribution

**Fig 2:**
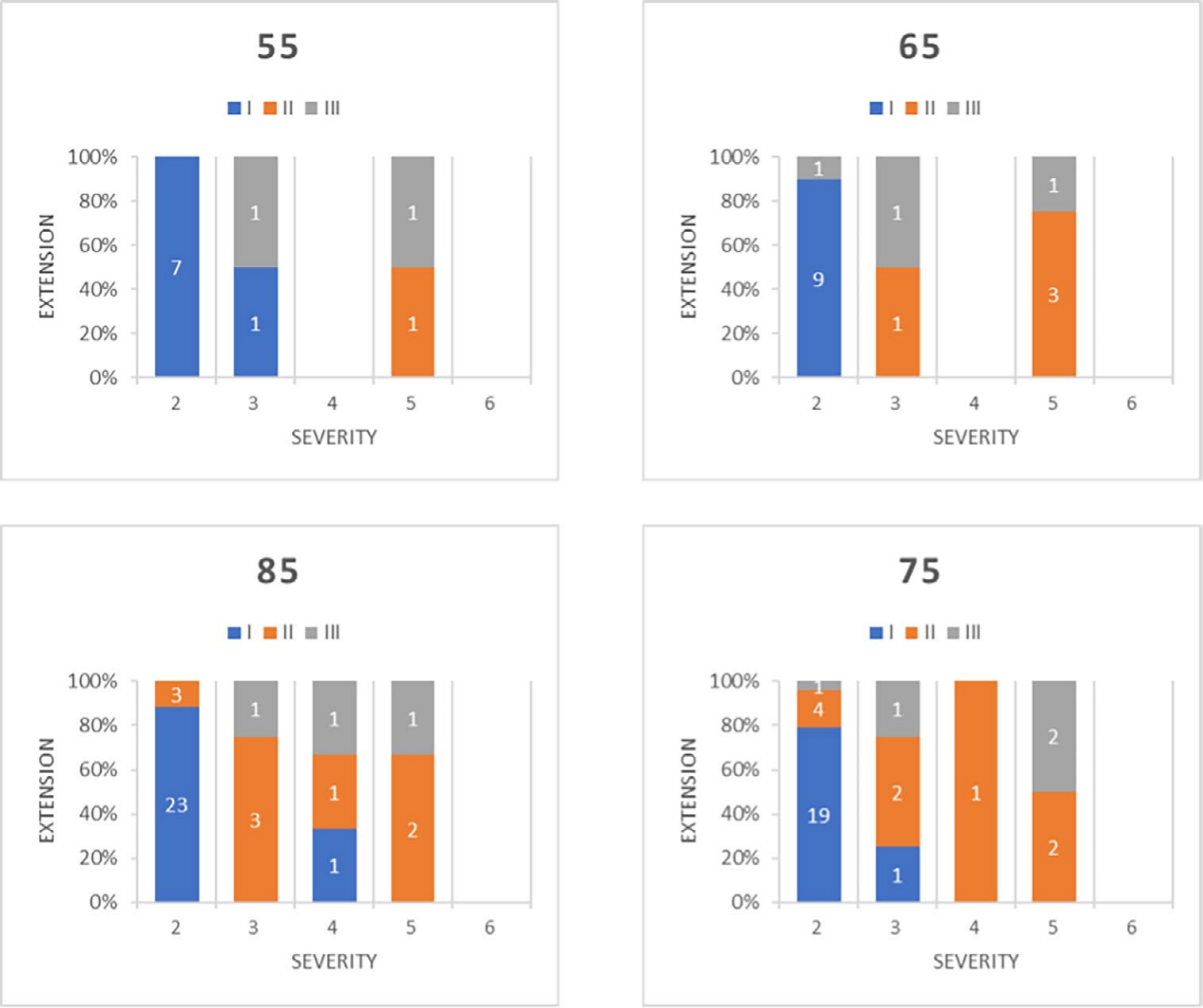
Distribution of HSPM on affected surfaces, modified index used.

Among the 1,035 children, 46 were found to have HSPM in 96 teeth. The prevalence was determined to be 4.4% at the child level and 2.31% at the tooth level. The mandible (71.9% (n = 69)) was affected in most of the cases, while the maxilla was less affected (28.1% (n = 27)). Affected children had, on average, 2.17 ± 0.99 teeth exhibiting HSPM. Most of the cases (69.8% (n = 67)) were considered mild (not rated higher than 2), with 12 of the cases being rated 3, 4 of the cases were rated 4 and 13 of the cases were rated 13. When looking at the affected surface area of the teeth, most of the cases were seen as not exceeding 1/3 of the tooth’s surface (63.5% (n = 61)), 23 of the cases were rated as between 1/3 of the tooth’s surface and 2/3 of the tooth’s surface with 12 of the cases were rated as exceeding 2/3 of the tooth’s surface (Fig 2).

### Association between HSPM and MIH

Among the 1,035 children examined, 166 (16.0%) had at least 1 erupted first permanent molar, and 6 of these (3.6%) presented with MIH. Within this subgroup, a statistically significant association was found between HSPM and MIH. MIH was more frequently observed among children with HSPM than among those without HSPM (*p* < 0.001).

Since MIH could only be assessed in children with at least one erupted first permanent molar, the multivariable logistic regression analysis was repeated in this subgroup. In the model constructed using the Forward (Wald) method, MIH was found to be significant and was shown to be independently associated with HSPM (OR = 49.667; 95% CI: 6.950–354.910; *p* < 0.001) (Table 2).

**Table 2:** Association between HSPM and MIH

Variable	B	SE	Wald test statistic	*p* value	OR exp (B)	95% CI
MIH (present/absent*)	3.905	1.003	15.150	< 0.001	49.667	6.950–354.910
Constant	–3.905	0.583	44.852	< 0.001	0.020	–
*Reference category, **Multivariable logistic regression analysis, B: regression coefficient, SE: standard error, OR: odds ratio, Exp(B): exponentiated regression coefficient (odds ratio), CI: confidence interval.						

### dft and HSPM Relationships

There seems to be no significant relation between dft and HSPM (*p* = 0.767) (Table 3).

**Table 3:** Associated factors and HSPM

	HSPM			
	Yes N (%)	No N (%)	Total N (%)	*p* value
**Maternal diabetes**				
Yes	8 (17.4%)	115 (11.6%)	123 (11.9%)	0.343
No	38 (82.6%)	874 (88.4%)	912 (88.1%)	
TOTAL N (%)	46 (100%)	989 (100%)	1035 (100%)	
**Maternal hypertension**				
Yes	2 (4.3%)	53 (5.4%)	55 (5.3%)	1.000
No	44 (95.7%)	936 (94.6%)	980 (94.7%)	
TOTAL N (%)	46 (100%)	989 (100%)	1035 (100%)	
**Usage of vitamin D supplements during pregnancy**				
Yes	16 (34.8%)	217 (21.9%)	233 (22.5%)	0.063
No	30 (65.2%)	772 (78.1%)	802 (77.5%)	
TOTAL N (%)	46 (100%)	989 (100%)	1035 (100%)	
**Vitamin supplement usage during pregnancy**				
Yes	17 (37.0%)	276 (27.9%)	293 (28.3%)	0.244
No	29 (63.0%)	713 (72.1%)	742 (71.7%)	
TOTAL N (%)	46 (100%)	989 (100%)	1035 (100%)	
**Calcium supplement usage during pregnancy**				
Yes	5 (10.9%)	108 (10.9%)	113 (10.9%)	1.000
No	41 (89.1%)	881 (89.1%)	922 (89.1%)	
TOTAL N (%)	46 (100%)	989 (100%)	1035 (100%)	
**Other supplement usages during pregnanc**y				
Yes	4 (8.7%)	119 (12.0%)	123 (11.9%)	0.652
No	42 (91.3%)	870 (88.0%)	912 (88.1%)	
TOTAL N (%)	46 (100%)	989 (100%)	1035 (100%)	
**Fluoride usage during pregnancy**				
Yes	4 (8.7%)	76 (7.7%)	80 (7.7%)	0.776
No	42 (91.3%)	913 (92.3%)	955 (92.3%)	
TOTAL N (%)	46 (100%)	989 (100%)	1035 (100%)	
**Antibiotic usage during pregnancy**				
Yes	1 (2.2%)	59 (6.0%)	60 (5.8%)	0.513
No	45 (97.8%)	930 (94.0%)	975 (94.2%)	
TOTAL N (%)	46 (100%)	989 (100%)	1035 (100%)	
**Premature birth**				
Yes	5 (10.9%)	98 (9.9%)	103 (10.0%)	0.800
No	41 (89.1%)	891 (90.1%)	932 (90.0%)	
TOTAL N (%)	46 (100%)	989 (100%)	1035 (100%)	
**Birth weight (gr.)**				
Lower than 1500	0 (0%)	17 (1.7%)	17 (1.6%)	0.570
1500–2500	8 (17.4%)	140 (14.2%)	148 (14.3%)	
Higher than 2500	38 (82.6%)	832 (84.1%)	870 (84.1%)	
TOTAL N (%)	46 (100%)	989 (100%)	1035 (100%)	
**Breastfeeding**				
Yes	45 (97.8%)	948 (95.9%)	993 (95.9%)	1.000
No	1 (2.2%)	41 (4.1%)	42 (4.1%)	
TOTAL N (%)	46 (100%)	989 (100%)	1035 (100%)	
**Baby bottle usage**				
Yes	30 (65.2%)	576 (58.2%)	606 (58.6%)	0.432
No	16 (34.8%)	413 (41.8%)	429 (41.4%)	
TOTAL N (%)	46 (100%)	989 (100%)	1035 (100%)	
**Usage of supplements**				
Yes	16 (34.8%)	334 (34.0%)	350 (33.8%)	1.000
No	30 (65.2%)	649 (66.0%)	679 (65.6%)	
Missing	0 (0.0%)	6 (0.6%)	6 (0.6%)	
TOTAL N (%)	46 (100%)	989 (100%)	1035 (100%)	
**Febrile illnesses**				
Chickenpox	0 (0%)	7 (0.7%)	7 (0.7%)	0.829
Rubella	0 (0%)	1 (0.1%)	1 (0.1%)	
No	46 (100%)	980 (99.2%)	1026 (99.1%)	
Missing	0 (0.0%)	1 (0.1%)	1 (0.1%)	
TOTAL N (%)	46 (100%)	989 (100%)	1035 (100%)	
**Asthma**				
Yes	2 (4.3%)	27 (2.8%)	29 (2.8%)	0.379
No	44 (95.7%)	948 (97.2%)	992 (95.8%)	
Missing	0 (0.0%)	14 (1.4%)	14 (1.4%)	
TOTAL N (%)	46 (100%)	989 (100%)	1035 (100%)	
**High fever**				
Yes	1 (2.2%)	73 (7.5%)	74 (7.1%)	0.247
No	45 (97.8%)	899 (92.5%)	944 (91.2%)	
Missing	0 (0.0%)	17 (1.7%)	17 (1.6%)	
TOTAL N (%)	46 (100%)	989 (100%)	1035 (100%)	
**Diarrhoea**				
Yes	2 (4.4%)	26 (2.7%)	28 (2.7%)	0.352
No	43 (95.6%)	951 (97.3%)	994 (96.0%)	
Mİissing	1 (2.2%)	12 (1.2%)	13 (1.3%)	
TOTAL N (%)	46 (100%)	989 (100%)	1035 (100%)	
**Pneumonia**				
Yes	2 (4.3%)	31 (3.2%)	33 (3.2%)	0.657
No	44 (95.7%)	943 (96.8%)	987 (95.4%)	
Missing	0 (0.0%)	15 (1.5%)	15 (1.4%)	
TOTAL N (%)	46 (100%)	989 (100%)	1035 (100%)	
**Bronchitis**				
Yes	6 (13.0%)	170 (17.4%)	176 (17.0%)	0.574
No	40 (87.0%)	808 (82.6%)	848 (81.9%)	
Missing	0 (0.0%)	11 (1.1%)	11 (1.1%)	
TOTAL N (%)	46 (100%)	989 (100%)	1035 (100%)	
**Otitis media**				
Yes	9 (19.6%)	195 (20.0%)	204 (19.7%)	1.000
No	37 (80.4%)	781 (80.0%)	818 (79.0%)	
Missing	0 (0.0%)	13 (1.3%)	13 (1.3%)	
TOTAL N (%)	46 (100%)	989 (100%)	1035 (100%)	
**Nephrological diseas**				
Yes	1 (2.2%)	17 (1.7%)	18 (1.7%)	0.566
No	45 (97.8%)	960 (98.3%)	1005 (97.1%)	
Missing	0 (0.0%)	12 (1.2%)	12 (1.2%)	
TOTAL N (%)	46 (100%)	989 (100%)	1035 (100%)	
**Urinary tract disease**				
Yes	8 (17.4%)	160 (16.4%)	168 (16.2%)	1.000
No	38 (82.6%)	816 (83.6%)	854 (82.5%)	
Missing	0 (0.0%)	13 (1.3%)	13 (1.3%)	
TOTAL N (%)	46 (100%)	989 (100%)	1035 (100%)	
**Gastrointestinal diseases**				
Yes	3 (6.5%)	49 (5.0%)	52 (5.0%)	0.504
No	43 (93.5%)	928 (95.0%)	971 (93.8%)	
Missing	0 (0.0%)	12 (1.2%)	12 (1.2%)	
TOTAL N (%)	46 (100%)	989 (100%)	1035 (100%)	
	Median (Min–Max)	Median (Min–Max)		
dft	2.0 (0–6.0)	2.0 (0–6.0)		0.767


### HSPM and Associated Factors

HSPM and Associated Factors were presented in Table 3. None of the assessed prenatal, perinatal, or early-childhood factors showed a statistically significant association with HSPM. No significant relationship was found for maternal illness during pregnancy (*p* = 0.250), maternal diabetes (*p* = 0.343), maternal hypertension (*p* = 1.00), or maternal supplement use during pregnancy, including general supplements (*p* = 0.652), vitamin D (*p* = 0.063), calcium (*p* = 1.00), fluoride (*p* = 0.776) and other supplements (*p* = 0.244).

Similarly, perinatal factors such as premature birth (*p* = 0.800) and birth weight (*p* = 0.570) were not associated with HSPM. Postnatal feeding practices, including breastfeeding (*p* = 1.00) and baby bottle usage (*p* = 0.432), also showed no significant relationship.

No significant associations were found for supplement use during the first year of life (*p* = 1.00) or for common childhood illnesses occurring within the first year (*p* = 0.829). Childhood medical conditions – including asthma (*p* = 0.379), high fever (*p* = 0.247), pneumonia (*p* = 0.657), bronchitis (*p* = 0.574), otitis media (*p* = 1.00), nephrological diseases (*p* = 0.566), urinary tract infections (*p* = 1.00), and gastrointestinal diseases (*p* = 0.504) – were likewise not significantly associated with HSPM.

## DISCUSSION

The present study aimed to assess the prevalence of HSPM among children aged 3–5 years living in Aydın, Turkey, and to examine potential associated factors. The prevalence of HSPM was determined to be 4.4% at the child level and 2.31% at the tooth level, which is slightly lower than the pooled global prevalence reported in the literature, but remains within the reported international range.

In the present study, no statistically significant association was found between HSPM occurrence and sex, age, school type, or parental educational level, and this finding is broadly consistent with the results reported in many previous studies.^6,18,19,20,21,22^ These results suggest that the sociodemographic variables examined may not play a decisive role in the development of HSPM within the present study population. In the study^26^ conducted in Türkiye by Taşçı et al, these four variables were evaluated simultaneously, and only age was reported to be associated with HSPM; however, a large body of literature has demonstrated no significant relationship between HSPM and either age or sex.^6,19–22^ The meta-analysis^18^ performed by McCarra et al likewise highlighted that although age and sex are frequently analysed parameters in HSPM research, most studies have failed to show meaningful associations between these variables and HSPM.

Consistent with the findings of the present study, the absence of a significant association between HSPM and school type, parental educational level, and broader socioeconomic indicators has been repeatedly reported in the literature.^21,22,26^ Nevertheless, a study specifically examining maternal educational attainment reported a lower risk of HSPM among children of mothers with a bachelor’s degree, a finding that was attributed to higher levels of maternal health literacy and greater adherence to prenatal care practices.^1^ Taken together, these findings indicate that HSPM is more strongly related to biological and environmental mechanisms than to demographic characteristics. The repeated observation that sociodemographic variables are not significant across different populations supports the interpretation that these variables exert, at most, a secondary influence on the development of HSPM and reinforces the conclusions drawn from the present study.

A recent meta-analysis reported^18^ that the prevalence of HSPM varies widely across studies, ranging from 0% to 41%. The pooled global prevalence in this meta-analysis was estimated at 6.8%. One of the earliest investigations into HSPM prevalence, conducted in the Netherlands, reported a rate of 4.9%.^6^ Subsequent studies have documented prevalence of 14.12% in Australia,^21^ 9.5% in southwestern France,^10^ 11.4% in Saudi Arabia,^2^ and 18.5% in Brazil.^4^ In Turkey, research on HSPM prevalence remains limited. A study conducted in the province of Uşak identified a prevalence of 3.5%3, while another study in Trabzon reported a rate of 6.8%.^26^ In this context, the prevalence values obtained in the present study are consistent with both international and national findings, thereby contributing valuable data to the existing literature on HSPM among preschool children in Turkey.

The heterogeneity observed in HSPM prevalence across different countries and regions is likely attributable to a combination of methodological and environmental factors. Variations in diagnostic criteria and indices present a major source of inconsistency, complicating direct comparisons between studies. In the present study, the index recommended by the European Academy of Paediatric Dentistry (EAPD) was employed, supporting greater standardisation of diagnostic outcomes. Additionally, differences in age ranges and geographic or environmental exposures may also influence reported prevalence figures.

In the present study, HSPM lesions were observed more frequently in the mandibular second primary molars (71.9%) than in their maxillary counterparts. This finding is consistent with previous studies reporting a higher prevalence of HSPM in the mandible.^4,19,25^ Among the limited number of studies providing tooth-specific data, Amarante et al reported^4^ that HSPM most frequently affected tooth #85. The higher occurrence of HSPM in mandibular teeth has been attributed to the earlier onset of enamel mineralisation, prolonged exposure to systemic influences during the prenatal period, and earlier involvement in active occlusal function, resulting in greater exposure to masticatory forces. In addition, increased mechanical stress during early childhood, differences in the vascular supply of the mandible, and the occlusal positioning of mandibular teeth, which may facilitate earlier clinical detection of lesions, may further contribute to the observed prevalence differences. Nevertheless, some studies have reported no significant differences in HSPM distribution between the maxillary and mandibular arches.^6,21^ Moreover, one study identified tooth #55 as the most frequently affected tooth.^20^ Taken together, these heterogeneous findings suggest that arch- and tooth-specific distribution patterns of HSPM may vary according to age group, sample characteristics, and methodological differences across studies.

In the present study, the majority of HSPM lesions were classified as mild (69.8%), typically presenting as well-demarcated white or cream opacities, with less than one-third of the tooth surface affected (63.5%). These findings are consistent with previous reports indicating that HSPM commonly manifests as limited-extent, clinically mild lesions.^14,18,23^ According to the EAPD criteria, the patterns observed in this cross-sectional study correspond to the category of minimal involvement, suggesting that the lesions generally do not pose a substantial threat to the structural integrity of the tooth.

The predominance of mild severity observed in this study highlights the clinical importance of early diagnosis of HSPM. Early detection is beneficial in that it enhances diagnostic accuracy, reduces the need for invasive procedures, and facilitates the timely implementation of preventive strategies. However, it is important to note that moderate and severe cases may require more durable restorative options due to hypersensitivity and the inherent bonding limitations of hypomineralised enamel. Consistent with this view, Elfrink et al.^8^ emphasised that the optimal period for assessing HSPM is between 3 and 5 years, when enamel defects can be identified more accurately, and the risk of misclassification due to post-eruptive breakdown is reduced.

In this study, the significant association identified between HSPM and MIH suggests that these two enamel developmental defects may share similar developmental pathways and common etiological mechanisms. Luppieri et al.^16^ likewise reported that a substantial proportion of children presenting with MIH also exhibited HSPM. The literature further indicates that HSPM may act as an early marker for MIH and that both defects are influenced by the same periods of biological stress during tooth development.^12,14,18^ A recent meta-analysis also demonstrated a significant positive association between MIH and HSPM^28^. However, although the presence of HSPM may increase the likelihood of subsequent MIH development, the absence of HSPM does not exclude the diagnosis of MIH.^7,12,19^ On the other hand, it should be noted that MIH is typically diagnosed following the eruption of the first permanent molars, usually around the age of six. Therefore, the inclusion of children aged 3–5 years in the present study resulted in a limited number of participants with fully erupted first permanent molars. This may have influenced the strength and interpretation of the observed association between HSPM and MIH. Accordingly, this issue represents an important methodological limitation of the study, and the findings should be interpreted with caution. Further studies involving older age groups with fully erupted permanent dentition are needed to provide more robust evidence regarding this association.

In the present study, no statistically significant association was observed between the presence of HSPM and dft scores. Although the increased organic content, porosity, and reduced mineral density of hypomineralised enamel are biologically expected to heighten susceptibility to caries,^12,21^ the findings of the current study did not support such a relationship. Variations in sample characteristics, age groups, oral hygiene habits, and diagnostic methods across studies may contribute to inconsistencies in the reported outcomes. Moreover, the limited surface involvement typically observed in HSPM-affected teeth, together with the predominance of mild lesions, may delay the clinical manifestation of caries risk^18^. These findings underscore that early-age screening is clinically valuable not only for the detection of existing enamel defects but also for anticipating caries risk and facilitating the timely delivery of preventive measures.

Given the cross-sectional design of the present study, a causal relationship cannot be established; rather, only potential associations between prenatal, perinatal, and postnatal factors and HSPM were examined. However, it has been reported in the literature that several potential etiological factors – such as prematurity, low birth weight, febrile and respiratory illnesses, and antibiotic intake – may contribute to the development of HSPM.^14,18^ Nevertheless, the findings across studies remain inconsistent, supporting the notion that HSPM is not attributable to a single determinant but results from multifactorial biological mechanisms.

In the present study, no statistically significant associations were identified between HSPM and prenatal factors (maternal illness, hypertension, diabetes, history of infection/fever, antibiotic and vitamin use) or perinatal conditions (prematurity, low birth weight). Although some studies have suggested that maternal systemic conditions during pregnancy may influence enamel mineralisation,^8,24^ others have reported no such relationship.^4,21^ Similarly, while perinatal complications have not always been linked to enamel hypomineralisation highlighted that prematurity and low birth weight may serve as possible risk factors.^14,21^ These discrepancies may be explained by variations in exposure severity, duration, timing, and maternal–foetal biological response, in addition to methodological heterogeneity across studies. The indirect and complex nature of perinatal influences may also hinder the detection of consistent associations in different populations.

With regard to postnatal variables (febrile episodes, respiratory infections, recurrent illness, antibiotic use, otitis media, pneumonia, feeding practices and breastfeeding duration, gastrointestinal and urinary diseases), no significant associations were observed. Although some reports have suggested that recurrent infections, fever, and antibiotic exposure could adversely affect enamel development,^12,18^ others have failed to demonstrate this link^21^. One possible explanation is that postnatal illnesses may be mild, promptly treated, or may not coincide temporally with the critical window of enamel mineralisation.

A major strength of the present study is the large and representative sample, along with the rigorous examiner calibration for HSPM diagnosis. Conducting examinations among children aged 3–5 years aligns with the recommended age window for accurate detection of HSPM, thereby reducing the risk of underdiagnosis or misclassification due to post-eruptive changes and enhancing the reliability of prevalence estimates.^8^

However, the cross-sectional design limits the ability to infer causality between etiological factors and HSPM. Moreover, data collection based on parental questionnaires introduces a potential recall bias, which may compromise the validity of past medical information. Another limitation relates to the school‐based setting of the clinical examinations, which may have imposed restrictions on lighting and moisture control, thereby increasing the likelihood that mild defects were overlooked. Finally, the absence of statistically significant associations should not be interpreted as evidence of no effect; rather, it reflects limitations in exposure characterisation (timing, severity, duration) and the inherent constraints of the study design.

Therefore, future research should involve prospective longitudinal cohort designs, incorporating objective biomarkers and integrated clinical–radiographic–molecular assessments to more clearly elucidate the etiological pathways underlying HSPM.

This study demonstrated that the prevalence of HSPM among 3–5-year-old children in Aydın is slightly lower than the pooled global prevalence but falls within the range reported in previous international and national studies. In this study, HSPM was not significantly associated with sex, age, or socioeconomic variables; however, it was observed more frequently in mandibular molars. A significant association between HSPM and MIH was observed, which suggests a potential link between these two enamel developmental defects; however, further longitudinal studies are needed to better clarify the nature of this relationship and their possible shared underlying mechanisms.

### Acknowledgements

The authors gratefully acknowledge all participating children and their parents/guardians for their time and cooperation. We also thank the administrators and staff of the participating kindergartens/clinics in Aydın, Turkey, for their assistance in facilitating data collection. We sincerely thank Dr Hakan Öztürk for his substantive contribution to the study through statistical analysis and guidance on data interpretation.

#### Statement of ethics

Ethics committee approval for this study was obtained from the Non-Interventional Clinical Research Ethics Committee of the Faculty of Dentistry, Adnan Menderes University, Aydın (AADÜDHF 2023/07). It was conducted in accordance with the World Medical Association’s Declaration of Helsinki. As the study was to be conducted in schools, official permission was obtained from the Aydın Provincial National Education Research Planning Board prior to the study. All participants and their parents/legal guardians were informed, and consent forms were signed.

#### Funding sources

This study was not supported by any sponsor or funder.

#### Conflict of interest statement

The authors have no conflicts of interest to declare.

#### Data availability statement

The data that support the findings of this study are available from the corresponding author upon reasonable request.
